# Watcher: Cloud-Based Coding Activity Tracker for Fair Evaluation of Programming Assignments

**DOI:** 10.3390/s22197284

**Published:** 2022-09-26

**Authors:** Youngpil Kim, Kyungwoon Lee, Hyunchan Park

**Affiliations:** 1 Department of Information and Telecommunication Engineering, Incheon National University, Incheon 22012, Korea; 2 School of Electronics Engineering, Kyungpook National University, Daegu 41566, Korea; 3 Division of Computer Science and Engineering, Jeonbuk National University, Jeonju 54896, Korea

**Keywords:** online learning, coding activity, cloud platform, Web-IDE

## Abstract

Online learning has made it possible to attend programming classes regardless of the constraint that all students should be gathered in a classroom. However, it has also made it easier for students to cheat on assignments. Therefore, we need a system to deal with cheating on assignments. This study presents a Watcher system, an automated cloud-based software platform for impartial and convenient online programming hands-on education. The primary features of Watcher are as follows. First, Watcher offers a web-based integrated development environment (Web-IDE) that allows students to start programming immediately without the need for additional installation and configuration. Second, Watcher collects and monitors the coding activity of students automatically in real-time. As Watcher provides the history of the coding activity to instructors in log files, the instructors can investigate suspicious coding activities such as plagiarism, even for a short source code. Third, Watcher provides facilities to remotely manage and evaluate students’ hands-on programming assignments. We evaluated Watcher in a Unix system programming class for 96 students. The results showed that Watcher improves the quality of the coding experience for students through Web-IDE, and it offers instructors valuable data that can be used to analyze the various coding activities of individual students.

## 1. Introduction

The COVID-19 pandemic has had a drastic impact on several industries across the world. University education is among the worst-affected sectors and has undergone significant changes [1]. Many offline-oriented classes were forcibly moved to online, non-face-to-face platforms, and both students and instructors had to make intricate adjustments [2,3]. Due to the high-speed internet and cloud computing infrastructure, we inferred that online classes are technically feasible much better than we expected and that offline–online hybrid classes are possible in the post-pandemic period.

Online classes have different aspects compared to traditional classrooms. First, the location of classrooms is not fixed; so, students can take courses anywhere they want. This increases the possibility of cheating because the illegal actions of students cannot be directly monitored by instructors when participating in online classes from within their private rooms [4,5]. For example, in the case of computer programming assignments, the fundamental and primary task of the students is to write the given assignments by themselves. However, in online classes, students can easily share their source code with each other, search the Internet for the assignment results [6,7], and submit the assignment through “copy and paste”. If left unattended, it adversely affects other students who wish to attend the class faithfully and undermines fairness. Most importantly, cheaters will not be able to improve their own learning abilities. Second, students can submit programming assignments via email or file upload. This is different from traditional classrooms that submit the assignments in the form of a hard copy [8]. In this case, the instructors need to compare the submission of the students one by one for a fair evaluation. On the other hand, when the assignments are collected as digital files, the assignment assessment becomes much easier than in traditional classrooms. This raises interest in the automatic assessment [9,10] that evaluates programming assignments automatically using software programs. The major approaches to the automatic assessment with a fair evaluation can be divided into plagiarism detection on the submitted source code and the activity tracking of the students writing the source code. Different from the plagiarism detection [11,12,13,14] that has been widely studied for many years, monitoring the coding activity [15,16,17] of students has not been sufficiently investigated.

We present the Watcher system, which provides an automated cloud-based software platform for impartial and convenient online programming hands-on education. Mainly, we focus on collecting and monitoring coding activities during online programming education. One of the use cases of coding activity can be plagiarism detection, and Watcher can dramatically reduce the burden of data collection for an instructor who needs to detect plagiarism manually. For Watcher, it is assumed that there is at minimum a computer with an Internet connection and no problems with using a web browser. Students should have at least experience using an IDE. The instructor should be familiar with the use of development environment tools in Linux and with the software tool that can compare and analyze the automatically collected coding activity data. There are no additional assumptions except the above.

The three distinguishing features of Watcher are as follows. First, Watcher provides students with a consistent and fair programming environment through the Web-IDE. Second, Watcher provides valuable coding activity history data to determine whether a source code is plagiarized, even if it is short. Third, Watcher’s instructors can remotely manage and evaluate the hands-on programming development environments and quickly investigate suspicious coding activity through automatically collected code snapshots. It is also possible to give advice or warn students through real-time feedback.

The main contributions of this paper are as follows:

- We design and develop Watcher, a cloud-based system for online programming classes that can mitigate concerns about cheating on programming assignments.
- Watcher offers convenience and accessibility for both students and instructors by providing hands-on programming development environments and reporting on the coding activities of students.
- We evaluate the prototype of Watcher in the Unix system programming class with 96 students. The results show that Watcher successfully provides data for analyzing students’ diverse coding activities on programming assignments while providing a convenient and fair programming environment.

The remainder of this paper is organized as follows. Section 2 describes the related studies. Section 3 explains the design considerations and the details of Watcher architecture and behaviors. The effectiveness and performance of Watcher are given in Section 4, and Section 5 provides the conclusions of the study.

## 2. Related Work

Watcher offers isolated programming environments (i.e., student instances) for students on private cloud environments and automatically collects the coding activities of the students from the environments to prevent or detect cheating on programming assignments in online classes. This is necessary for recent online classes, different from traditional classrooms, as the possibility of cheating increases without the face-to-face monitoring of instructors [4,5,6,7]. Previous studies on such topics (i.e., detection of cheating on programming assignments) have mostly focused on detecting plagiarism in the students’ source code [12,13,14,18,19]. This is because the common approach to detect plagiarism in programming assignments is to compare the submitted assignments of students in the form of a hard copy in traditional classrooms. As many classes started to be held online because of the COVID-19 pandemic, different approaches have been adopted such as automatic assessment, analyzing student behavior, and tracking the coding activity of students. This section describes previous studies with different approaches for preventing or detecting cheating on programming assignments including traditional plagiarism detection.

Many studies on plagiarism detection aim to evaluate the similarity between the submitted source code of students, which can be categorized into different methods such as attribute-based and string-based [11]. For instance, Novak et al. proposed Dolos [13], a plagiarism detection tool for programming assignments by adopting a token-based algorithm. Although Dolos offers high accuracy in plagiarism detection compared with other techniques, the evaluation results show that the accuracy varies depending on the programming language. Similarly, Karnalim et al. introduced an assessment submission system to provide formative feedback on plagiarism and collusion. The system analyzes the similarity between the submitted assignments based on information retrieval and provides similarity reports to corresponding students and lecturers. However, the limitation of such a submission-based system is that the result of cheating can only be found after the assignment has been completed.

On the other hand, other studies suggested detecting similarity based on student behavior [12,19]. Hellas et al. [19] highlighted the limitation of the coarse-grained edit data utilized in previous studies and presented a coding activity-based plagiarism detection system that can be applied to take-home exams. Another study [12] proposed a plagiarism detection tool called BPlag, which overcomes plagiarism-hiding transformations that modify the original source code to hide the act of plagiarism. Although the study achieved high robustness and accuracy in plagiarism detection, it is limited to Java programming assignments. Thus, additional implementation efforts are necessary to integrate the technique into different programming languages.

In addition, there was an attempt to adopt machine learning techniques to detect cheating of students in programming tests [14]. The proposed technique utilizes a random forest model to predict each student’s score based on the previous results from the exercises given in the classes. When a student’s score is higher than the predicted score from the random forest model, the student can be suspected of cheating on the test. Even though the proposed technique offers high accuracy in detecting test cheating compared with previous techniques, it requires pre-processed data that consist of questions that contain the same knowledge points as that of the test questions to construct a random forest model. On the other hand, our technique does not require pre-processed data for detecting test cheating, as we record runtime statistics of students’ coding activity.

The proposed technique in this paper can examine whether students cheat on a programming assignment by tracking their coding activities. Several studies [15,16,17] have proposed the monitoring of students’ coding activities based on a similar approach. One study [17] developed a dashboard that enables self-reflection on coding activities to reflect students’ learning processes. Similarly, a research work [15] suggested a server–client assistance system to collect, store, and monitor programming activity by using logs. The proposed technique is implemented as the plug-in of a well-known IDE, Eclipse. Another study [16] presented DevActRec, an IDE-based tool to collect coding activity information for enterprise software developers in a non-intrusive and privacy-preserving manner. Even though previous studies [15,16,17] have implemented techniques to collect information on coding activity, they mostly run on a local computer collecting the runtime coding information. This may limit scalability when the number of students increases. On the other hand, Watcher runs on cloud environments; thus, it adopts the advantage of cloud environments such as flexibility and scalability.

An automated assessment tool, DSLab [20], has been introduced for online distributed programming, which is similar to the cloud-based architecture of Watcher. DSLab is developed for evaluating programming assignments wherein students submit their work via web interfaces to a front-end. Then, the back-end (LSim) evaluates the work by comparing the instance states of the instructor. DSLab only offers results of the assessment of programming assignments (correct or incorrect). However, Watcher can provide more detailed information by collecting the coding activities of the students. Furthermore, Watcher offers a student instance for every student to construct isolated execution environments that allow students to store their coding activities independently and safely.

Table 1 summarizes the previous studies relevant to Watcher. We compare the studies in terms of the research goal and characteristics to our technique, Watcher. First, we investigate whether the previous studies are able to (1) detect cheating on programming assignments, which can provide fair evaluation in programming assignments. As programming assignments can be conducted with any programming language and the length of the source code can vary, we also examine the techniques proposed in the previous studies that are (2) independent of programming languages and (3) applicable to the short-length programs. Next, we explore the fundamental architecture of the previous studies as to whether they are (4) based on cloud environments and (5) provide a single virtual machine (VM) for each student. At last, we list the integrated development environments that the previous techniques support. To the best of the author’s knowledge, Watcher is the first work that adopts cloud-based execution environments for programming assignments and can collect the coding activities of students.

## 3. Our Solution: Watcher

This section describes the design of Watcher. The design considerations are first explained, and the overall structure is presented. A detailed explanation of the operation of the system is also given in this section.

### 3.1. Design Considerations

We considered two main points when designing Watcher: isolation and convenience. The isolation can be achieved by providing each student with a high-level isolated execution environment. The students must possess isolated execution environments for programming assignments. Typically, execution environments include fundamental computing resources such as CPUs, memories, and storage volumes. If high-level isolation is not achieved, a student’s work may be exposed to other students, or one student’s programming mistake may destroy other students’ codes or execution environment. In cloud platforms, the execution environments are provided by public or private cloud providers in the form of manageable computing resources (i.e., VMs), and multiple users can share and access these resources safely. As Watcher runs on cloud-based execution environments, each student in Watcher possesses an independent VM referred to as a “student instance”. Furthermore, each student has complete control of a provided student instance. In the case of an execution environmental problem in the student instance, the owner student can directly check and resolve it by soft rebooting.

For convenience purposes, Watcher provides an easy way to collect programming activities at a low cost. The collection of data for programming activities is not simple because it requires data sharing between the isolated execution environments of the students. Additionally, it is a time-consuming and tedious job because the number of data sources increases with the number of students. If student instances are deployed in the distributed computers, the characteristics of physical networks should also be considered. Thus, an easy and low-cost method for collecting programming activities is required. Watcher enables transparent and efficient inter-instance communications between “student instance” and “master server instance” by introducing a virtual network. A feasible solution is to place all instances that should participate in communication in the same virtual network. The instances in the same virtual network can exchange data in a consistent and organized way. Due to the presence of a virtual network, we do not care about physical network configurations, topologies, or capacities. In addition, Watcher automatically manages coding activity data by collecting the coding activity logs from every student instance and sending them to a master server. Thus, instructors need not care about complicated network configuration and gathering coding activities; they can take advantage of their free time to enhance educational quality.

### 3.2. Architecture

**Figure 1:**
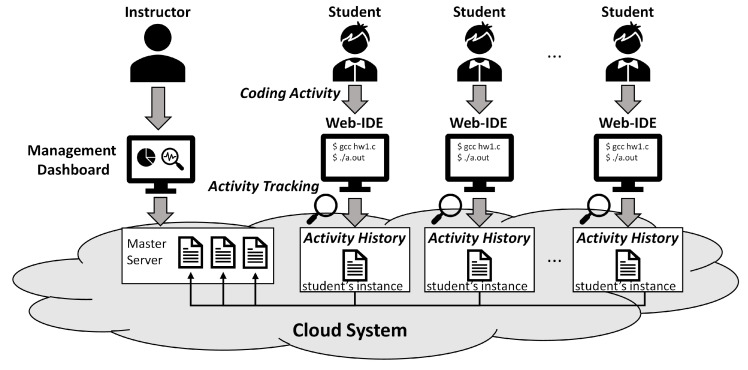
Overall architecture of Watcher.

Figure 1 shows the overall architecture of Watcher. Watcher deploys multiple instances on a virtual network within a virtual private cloud. Watcher provides student instances and instructor instances to students and instructors, respectively.

#### 3.2.1. Student Instance

Watcher creates student instances for a class based on a ready-made VM image with the same SW configuration. The VM image includes two main components: The *Web-IDE (integrated development environment)* and *Watcher client* running on a Linux distribution such as Ubuntu (Current Watcher prototype is based on Ubuntu version 20.04). Both components run automatically when the student instance boots. After booting, in the early setup stage, student instance sets each student’s student ID to the instance’s hostname.

**Figure 2:**
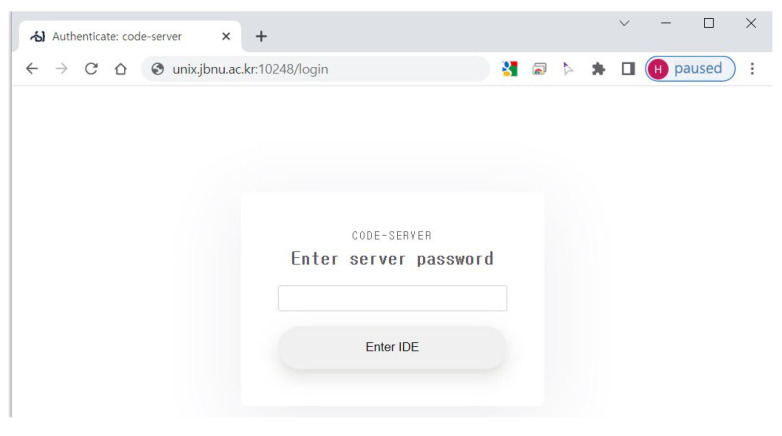
Login page of Watcher Web-IDE.

**Figure 3:**
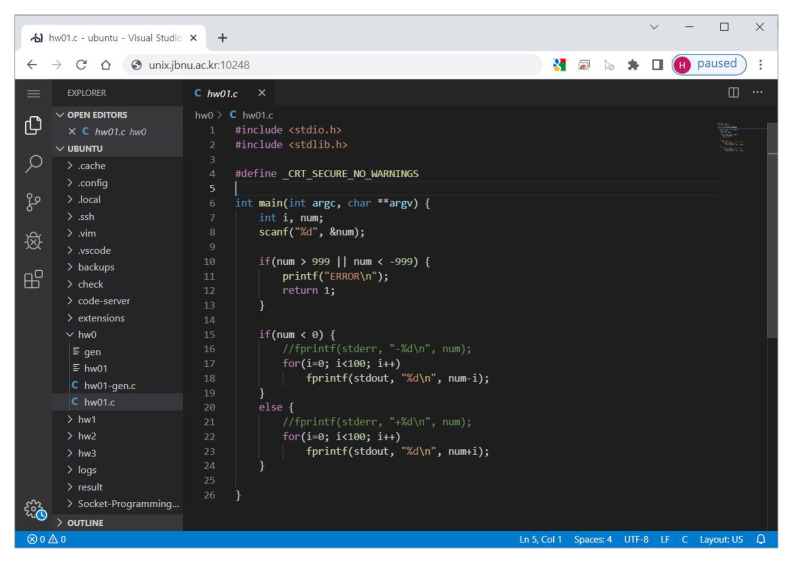
Code editing page of Watcher Web-IDE.

**Web-IDE** is a web-based IDE that provides a development environment to compile, run, and debug the code written by students through a web browser. Watcher Web-IDE is based on an open-source-based Visual Studio (VS) code server (https://github.com/coder/code-server. Accessed 16 September 2022), a widely used and customizable IDE. Students can conveniently compile, execute, and debug their code with the development extensions of VS-Code through the graphical user interface (GUI). Students can access their private Web-IDE through a web browser anytime and anywhere, as long as an internet connection is available (*immediate coding*). When accessing the Web-IDE, a password must be entered for security, and the password is automatically generated and provided to students when creating a student instance (see Figure 2). The Web-IDE provides the same kind of development and execution environment to all students, which increases the convenience of students and instructors (see Figure 3). The Web-IDE also provides an interactive terminal at the bottom for students to use conventional build tools such as “make”, “gcc”, and “gdb” in the student instance in real time. Students’ programs run in an independent execution environment (VM) as if they were running on a separate computer; so, they do not affect the execution of other students’ programs. The Web-IDE is set to track and record file changes every second. Thus, all data related to coding are safely and automatically stored (*auto-saving*). Watcher defines the automatically stored data as *coding activity*.

The **Watcher client** collects coding activity and sends it to the *master server*. The Watcher client monitors the working directory used in Web-IDE to track the updates of the files. When updated, the file is copied to the local temporary directory, referred to as the *local activity repository*; created for each file; and renamed to the current time. For example, the “abc.c” file is copied and renamed to “2022010135950.c” if the file is changed at 13:59:50 on 1 January 2022. Based on these operations, the change history for the files in the working directory can be stored together with the timestamp (*activity history*). The stored coding activities are continuously transmitted from a local repository (*activity repository*) to the master server. The local repository allows students to easily restore files even if they lose the files accidentally. At the same time, even if the activities of the master server are lost, they can be restored from the local backups of student instances.

#### 3.2.2. Instructor Instance

The instructor’s instance is configured by adding the following components on a general Linux distribution: a master server and a management dashboard. There is only one instructor instance per class. The virtual network IP addresses of the instances are predefined; thus, the Watcher client of the student instance can communicate with the instructor instance without any additional configuration. The master server receives coding activities delivered by the student instance and stores them in the *global activity repository*. The management dashboard helps monitor and analyze coding activities more conveniently.

**Master server** is a daemon process that always waits for a connection using a predefined IP address and port number. When a connection request is established from a Watcher client, a separate thread is created to receive the file that contains coding activities sent by the client. Then, the student ID and the file name are stored separately in the global activity repository. As we already set the student ID to a student instance’s hostname, the student ID can be obtained by receiving the hostname from the Watcher client. We can also know the specific file activity by the full path name received from the Watcher client. For example, if the student ID is “202012345”, the hostname of the student instance is “202012345”. Then, the file’s full path name saved in the global activity repository becomes “202012345/abc.c/2022010135950”. The middle string of “abc.c” stands for the target source file, and “2022010135950” indicates a specific activity at a given timestamp. We also consider the runtime errors. As the master server consists of a simple structure, restarting the daemon process in the case of errors is not expensive. Thus, we can easily maintain the master server clean and robust.

**Management dashboard** is a Web-based GUI that enables easy browsing and immediate analysis of data in the global activity repository. As the global activity repository is formed in a general directory tree structure, it is not difficult for an instructor to directly access and examine an instructor instance. Thus, it is intended to enhance the convenience of the instructor by providing tools to check students’ activities more conveniently and to analyze activities easily through the management dashboard. The analysis function provided by the management dashboard can be added according to the requirements. Basically, we can check the total size of activities for a specific assignment file, a graph of the size change of activities, the total execution time for a specific assignment, and the pattern of execution time. More complex analysis can be performed by directly copying the activity files.

### 3.3. Watcher Behaviors

**Figure 4:**
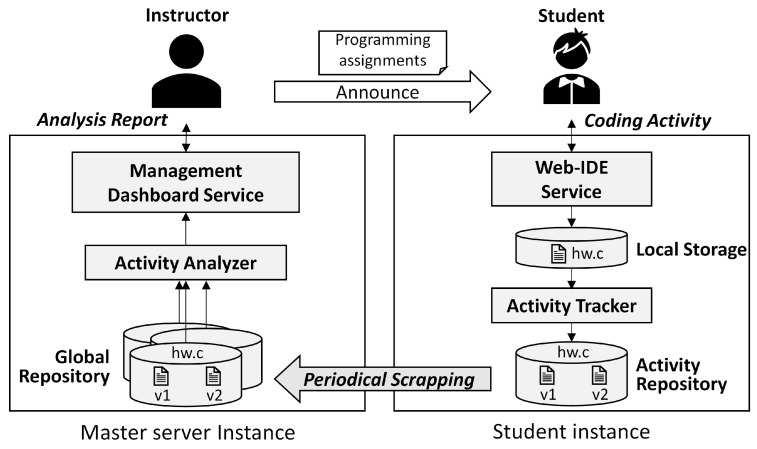
Watcher behavior.

This section describes the overall flow of Watcher operations in Figure 4 based on the components described in the previous subsection.

**Watcher tracking behavior.** The action starts with the coding activity of a student in the Web-IDE. The Web-IDE automatically updates the source code file in local storage when the coding activity stops for more than 1 s. At the same time, the Watcher client in a student instance monitors the source code file changes in the local storage at 0.5-s intervals. On a change, the updated file is copied to the reserved directory with the reserved file name in the local activity repository, and the updated file name is set as the combination of the current date and time. Then, the updated file is instantly transmitted to the master server. The aforementioned operations are referred to as *Watcher tracking behavior*. Watcher tracking behavior is continuously performed in a student instance.

The main issue with Watcher tracking behavior is how to reduce the amount of CPU consumption due to continuous monitoring operations. This is because the monitoring process of the Watcher client continuously utilizes the CPU in the student instance. To address this issue, we minimized the overhead of finding the updated file in a specific directory by sorting in the order of recent modification. Thus, the CPU utilization of the file searching process did not exceed 1%. As the source files produced from the coding activity are sufficiently small to be cached, the overhead of I/O operations is also negligible.

**Watcher analyzing assistant behavior.** Once the master server receives the updated file from the Watcher client, it stores the file in the global activity repository. Then, the instructor can analyze the activities for each assignment directly or by accessing them through the management dashboard. Basically, Watcher collects a series of snapshots of source code and various metadata related to coding activities, such as the number of coding activities, timestamps, file sizes, etc. In Section 4.5, we show how these data help analyze students’ coding activities.

## 4. Evaluation

We implemented a Watcher prototype in a private cloud environment based on Openstack version Ussuri (https://www.openstack.org/software/ussuri/. Accessed 16 September 2022) and adopted it to the major classes in the computer science department. First, we provided a questionnaire to the students; we analyze the results in Section 4.4. Then, we also evaluated whether the collected coding activity data were effective in analyzing students’ coding activity through real cases in Section 4.5.

### 4.1. Class Information

We used Watcher in a Unix system programming class in the fall semester of 2020 at JBNU (Jeonbuk National University) when the spread of COVID-19 was severe. A total of 96 students took the class. It should be noted that 56 students responded to our questionnaires from the class of 96 students. This class was an online hands-on class wherein various system programming assignments were performed individually.

### 4.2. Description of the Survey

The questionnaires consisted of six multiple-choice questions and four long-answer questions. We used Google Forms to construct the questionnaires and receive students’ responses. Note that the contents of choices for each question are described with each evaluation result. We provided the access link for the survey and allowed students to have time to respond to the questions thoroughly for a week. Most of the students submitted the responses in three days. We summarized the survey results in a spreadsheet document file and reorganized them as graphs in Section subsec:question.

We designed questions to achieve the three following goals and constructed two to four questions for each goal.

- To investigate the opinion of students with Watcher in terms of effectiveness for detecting suspicious coding activity (Section 4.4.1);
- To ask the experience with Watcher providing functionalities such as auto-saving and immediate coding (Section 4.4.2);
- To examine the user-experience of Watcher in terms of performance (Section 4.4.3).

As a result, we received complete responses from 58% of students in the survey period. This is because the survey was not mandatory for students. Furthermore, it was challenging to encourage students to participate in the survey because we only informed students of the survey in non-face-to-face forms (e-mail and messenger) due to COVID-19 circumstances.

### 4.3. Validity and Reliability

**Construct validity:** To ensure the purpose of the question, we first gave students similar but different questions to examine if students’ answers were consistent. Moreover, we offered both multiple-choice and long-answer questions, allowing students to describe their detailed opinions on each question. We can clarify that the intended meaning of each question is not misinterpreted by comparing the answers from the multiple-choice question and the corresponding long-answer question. Finally, we kept the number of questions to less than 10 to enable students to focus on responding to the questionnaires. This prevents students from answering randomly to the questionnaires if they do not have enough time to respond to many questions.

**Internal validity:** The survey proceeded for a week, and all students finished it before their grades were out. Thus, students could respond to the survey without being affected by their grades, i.e., they could respond the survey based on their experience in the class without too-positive or too-negative feelings about their grade. Next, we maintained anonymity in the questionnaires to allow students to describe their experience with Watcher straightforwardly.

**External validity:** It may be difficult to argue the generality of the survey result. However, the goal of this questionnaire is to show the applicability of Watcher to online programming assignments. Thus, we divided the answers to each multiple-choice question into five categories depending on the degree of positive or negative experience with Watcher. Then, we presented the percentile of each answer, such as 96%, to show the ratio that can imply the degree of generality of each question.

**Reliability:** For reliability, the study [21,22] described that the result should be the same if others conduct the same study. As our study applied Watcher to a single class and conducted a questionnaire for the students, it is challenging to evaluate reliability directly. Instead, we examine whether the Watcher can be repeatedly applied to different classes and evaluated with the same questionnaire. First, the Watcher system can be utilized for other classes repeatedly once it builds without errors because Watcher is based on VM, which supports easy migration to other platforms. Next, our questionnaires have been set in an electronic form and only deal with the effectiveness and functionality of the Watcher system, not the class quality. Thus, it is possible to evaluate Watcher in other classes using the same questionnaire. As long as the class carries out programming assignments, we expect it to show similar results to our study.

### 4.4. Survey Results and Analysis

#### 4.4.1. Is It Effective in Preventing Cheating?

**Figure 5:**
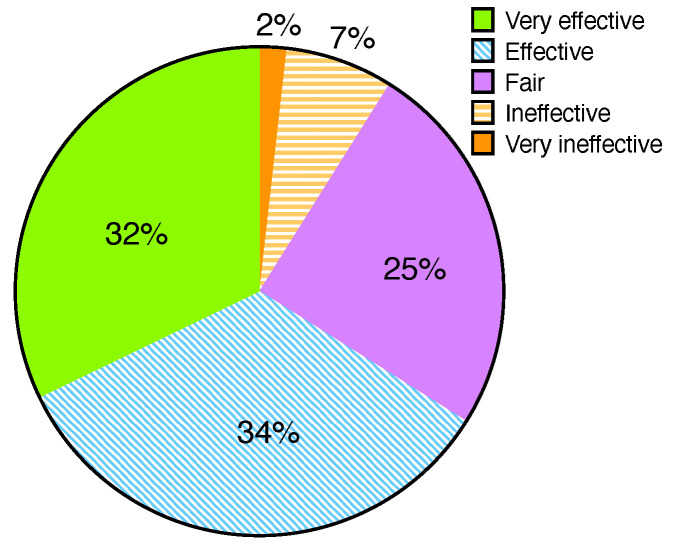
Effectiveness of Watcher.

First, we surveyed how students perceived Watcher in terms of prevention of cheating on programming assignments by allowing students to score the effectiveness of Watcher on a scale of one to five. A score of “five” indicates that the coding activity tracking of Watcher is significantly effective in preventing cheating on programming assignments, while “one” means ineffective. Figure 5 illustrates that 91% of the students in the class agreed that Watcher effectively prevents cheating. Only 9% of the students considered that it was not up to the mark. The reason for the ineffectiveness is that cheating students can exploit the system by allowing other students to write a program using different accounts to pretend to be the account owner. As Watcher uses the history of coding activity to detect cheating on programming assignments, it is difficult to identify cheating students when they are disguised as normal students.

#### 4.4.2. Convenience of Web-IDE Environment

In terms of the convenience of the Web-IDE offered by Watcher, we surveyed the coding experience of students. We asked students to rate the overall convenience of the Web-IDE on a scale of one to five. Then, we investigated the specific experiences of the students with two functionalities offered by the Web-IDE, which are auto-saving and immediate coding experience. Auto-saving means the programming results of the students are automatically stored in the Linux VM running Web-IDE. The immediate coding indicates that the students can start programming without additional installation or configuration.

**Figure 6:**
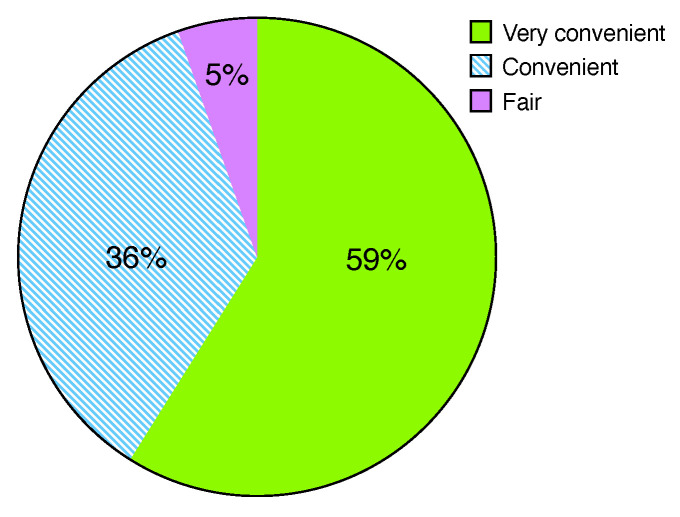
Overall convenience rate.

**Figure 7:**
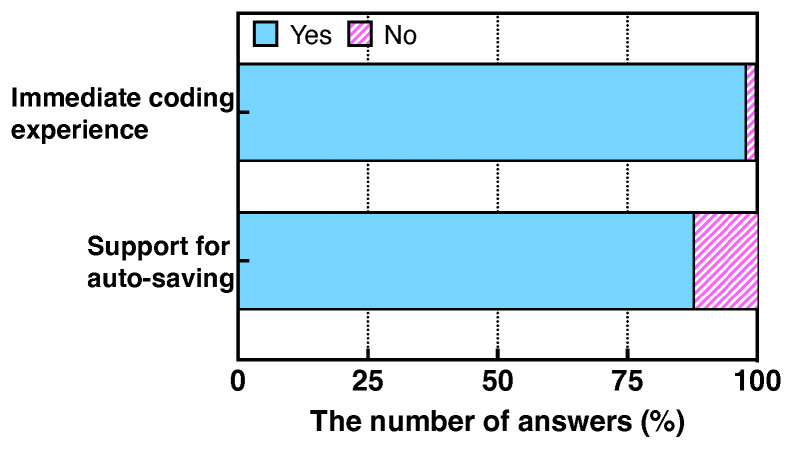
Benefits of the functionalities of Watcher.

Figure 6 shows that most students (i.e., 95%) answered that Web-IDE could be conveniently utilized in the class, while no student answered that Web-IDE is inconvenient. Only 5% of the students answered that the convenience of Web-IDE is fair. This result shows that Web-IDE benefits students who take programming classes to construct a programming environment. Figure 7 illustrates that 98% of the students found the immediate coding experience offered by Web-IDE to be highly beneficial. Only one out of fifty-six students responded that they had difficulty using Web-IDE because the student was unfamiliar with the IDE. Furthermore, 88% of the students answered that the auto-saving functionality is beneficial. Seven students responded that the auto-saving is inconvenient because they cannot roll back the programming history after programming results are automatically stored.

#### 4.4.3. Watcher Performance

As Watcher offers the Web-IDE on top of the native Linux VM for each student, it can cause additional performance overhead compared with that of the native Linux VM. Furthermore, Watcher is based on private cloud environments that can run multiple student instances on the same host servers, and the student instances can affect each other when students write programming assignments simultaneously. We investigated the experienced performance overhead caused by Web-IDE by surveying the students in the class.

**Figure 8:**
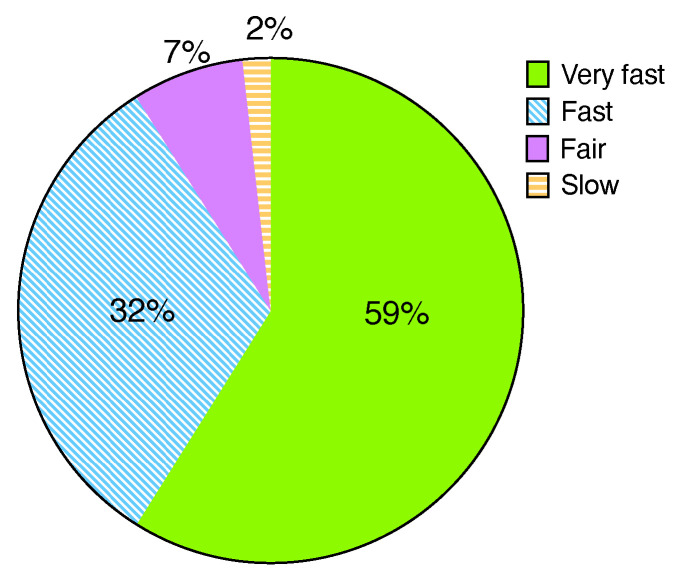
User-perceived performance.

Figure 8 depicts that only some students (2%) experienced slow performance when the memory size of the program increased. We can resolve this issue by calculating the students’ average memory usage and resizing the VM memory capacity. Most students (98%) responded that they did not face significant performance degradation during the class. These results show that Watcher can provide scalable performance even in a class of 50 or more students. We plan to extend Watcher to support multiple classes in parallel using a rack-scale test environment.

### 4.5. Analysis of Coding Activity Case

It is difficult to find the correct behavior from the given data. Instead, we examined a specific case and tried to use it as a hint for future analysis. We analyzed two students’ cases: high-scoring and low-scoring.

#### 4.5.1. High-Scoring Student Case

**Figure 9:**
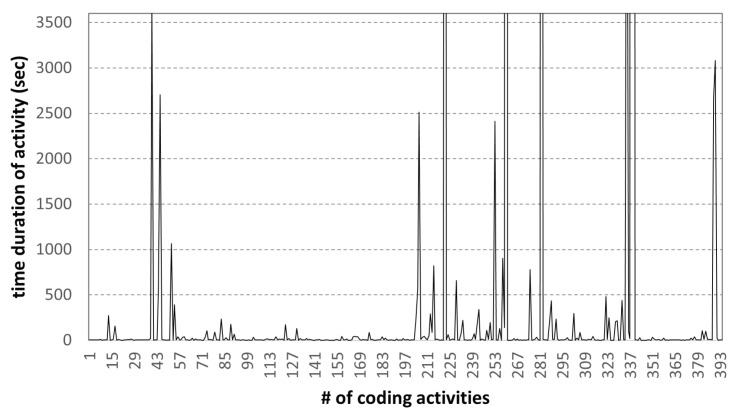
Time diff. of coding activities: high-scoring student.

Figure 9 shows the collected coding activities of the **high-scoring student** for one of the programming assignments. The assignment consisted of two source code parts: client and server.

In Figure 9, the x-axis represents the sequence number of coding activities and the y-axis is the duration of each coding activity. This result shows the change in activity time of 395 coding activity samples of best-scoring students. From the coding activity data, it can be observed that for a student with good coding skills, it took 153.66 h (approximately 6.4 days) to complete a given programming assignment. In addition to this, based on the history of coding activity data, we confirmed that it is possible to analyze the time of each coding activity. The high-scoring student spent an average of 23.4 min on 395 coding activities, from as short as 1 s to as long as 84.66 h (approximately 3.5 days). Such data will be useful as they can help analyze each student’s coding ability and type.

**Figure 10:**
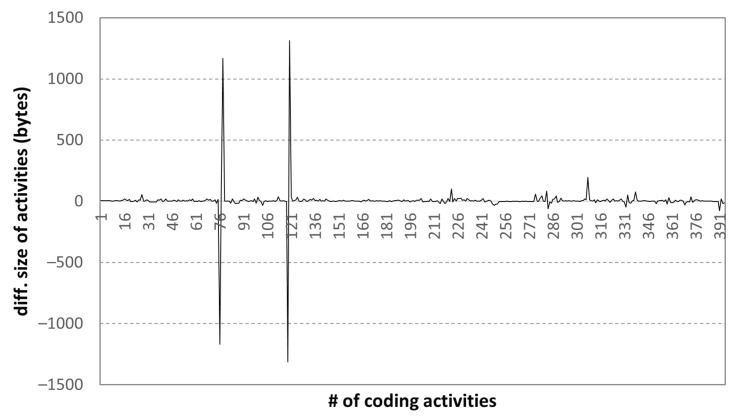
Diff. size of coding activities: high-scoring student.

Next, we examined the changes in the file size in coding activities; the results are shown in Figure 10.

The file size changes in the coding activity data indicate the source code modifications that were made. In general, while writing a source code, the file size increases steadily. However, if the long or redundant parts of the previously written source code are modified into short and efficient ones, the file size can be reduced. We can identify these normal aspects from the coding activity data. Moreover, from such cases, we observe two major revisions to the code that were written at the beginning of a programming task when the solution was unclear.

**Figure 11:**
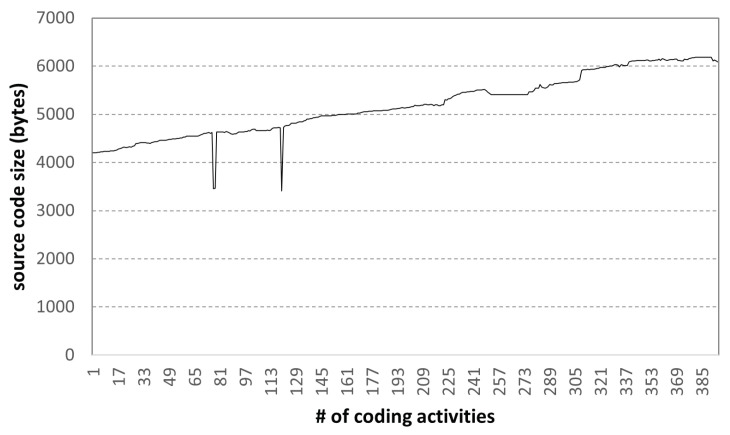
Code size variation in coding activities: high-scoring student.

Figure 11 shows the change in the size of the source code during 395 coding activities of a high-scoring student. We can observe two code revisions, which are mentioned in Figure 10; the code written at the beginning of about 4000 bytes is continuously written, and the size of the final code is approximately 6000 bytes. Based on the coding activity of excellent students, the size of the normal final programming assignment result can be roughly known, and this can be used as comparative data for cheating activity.

#### 4.5.2. Low-Scoring Student Case

**Figure 12:**
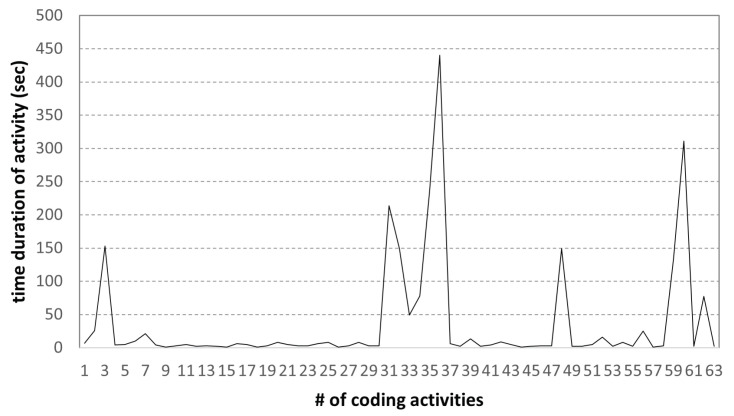
Time diff. of coding activities: low-scoring student.

**Figure 13:**
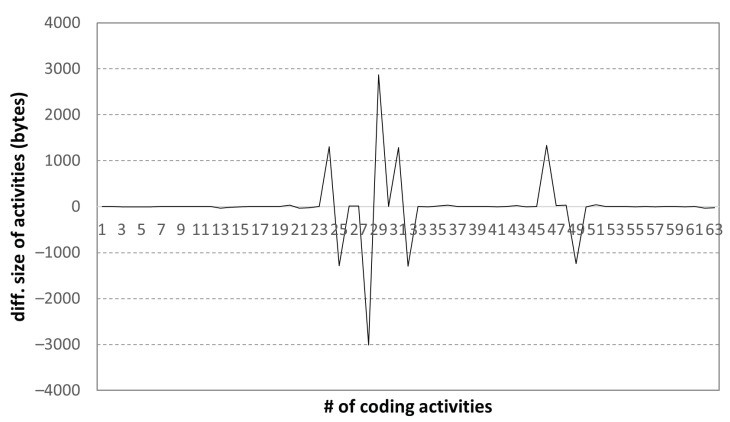
Diff. size of coding activities: low-scoring student.

In Figure 12, the x- and y-axes are the sequence number of coding activities and the duration of each coding activity, respectively. This result shows the change in activity time of 64 coding activity samples of low-scoring students. Unlike the high-scoring student, the student with low coding skills spent only 1 h writing a given programming assignment. Next, we examined the file size changes in coding activities; the results are shown in Figure 13.

It can be observed that the low-scoring student focused on the middle part of programming and made several code modifications. However, there was no significant change for the majority of the time.

**Figure 14:**
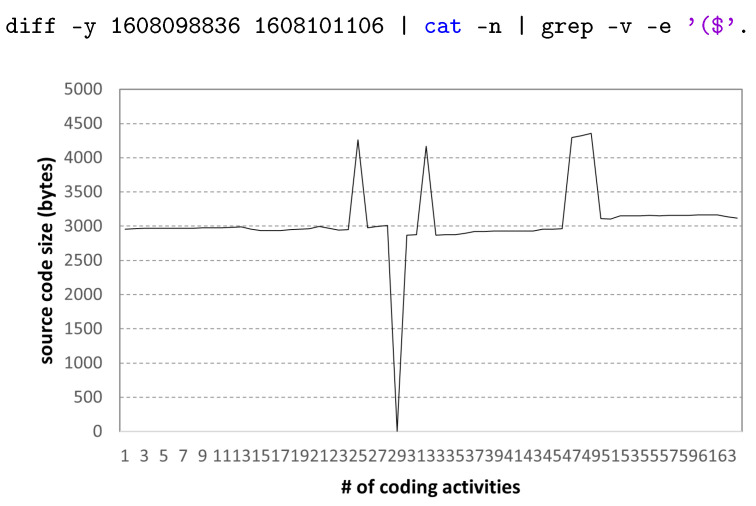
Code size variation in coding activities: low-scoring student.

Figure 14 shows the change in the size of the source code of the low-scoring student during 64 coding activities. We found that the size of the final code was almost identical to that of the original base code. To examine the reason, we compared the original code at coding activity 0 with the final code at coding activity 64. As Watcher stores snapshots of the intermediate source, we can obtain the compared results by the “diff” command. For example, we can compare the original coding activity file (1608098836) with the last one (1608101106) using the following command.

**Figure 15:**
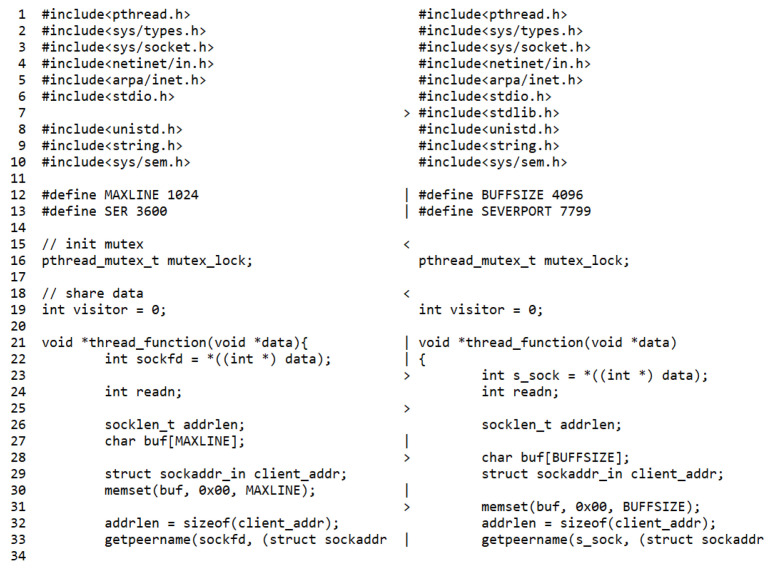
Code comparison example by “diff” original and final file: low-scoring student.

The original code file included 100 lines, and the final file of the low-scoring student had 118 (see Figure 15). In the final file, we observed no significant change in the original file except for adding blank lines, indents, comments, and a few lines of code. On the other hand, in the case of the high-scoring student (see Section 4.5.1), we observed that 291 lines of new code were written by adding three new functions and routines into the base code.

#### 4.5.3. Summary

Table 2 shows the summarized results of our coding activity data. We collected coding activity data for 66 students who submitted the assignment among the class of 96 students. Two groups of high and low grades were selected, and the total number of coding activities, total task execution time, file change size, and final source code file size were summarized accordingly. The submission rate was 68.75% because the assignment was challenging. We applied Watcher to all 13 programming assignments in the UNIX system programming class. We chose one of the challenging assignments for Watcher evaluation because it was expected that an attempt to cheat would occur on it.

Regarding the number of coding activities, high-scoring students showed at least 400 activities, while low-scoring students showed an average of several dozens. In the total task performance time, high-scoring students spent more than 70 h on average, while low-scoring students did not exceed 10 h. The final source code file size differed: high-scoring students showed 7000 or more bytes on average, whereas low-scoring students showed 2600 bytes on average, which is 30% of the high-scoring ones.

Interestingly, in the file size change, high-scoring students showed little difference between the maximum and minimum changes, while the low-scoring students showed an asymmetry of more than 700 bytes on average. Presumably, this is because the low-scoring students were unable to write new code completely; thus, parts of the code that did not correctly implement the assignment requirements were removed.

From the aforementioned Watcher data, we can gain insights into preventing or detecting students’ cheating behaviors. First, Watcher data represent the aspects of the regular coding activities. Regardless of whether the data are from a high-scoring or low-scoring student, the point is that these data describe normal coding activities, which are explainable. However, if the data are not explained well, Watcher helps determine the soundness of the coding activity by examining and comparing the snapshots of the source code. Second, in addition to detecting fraudulent behavior, Watcher can provide evidence for defending normal coding activities. As Watcher is based on metadata related to coding activity, it cannot perfectly detect cheating. This may lead to a situation where a student is unfairly suspected of cheating. However, because Watcher tracks all source code snapshots, it can be used as a basis for normal coding activity. In this way, Watcher can significantly contribute to the prevention of cheating and subsequently improve the fairness of coding activities.

## 5. Conclusions

This paper presents Watcher, a cloud-based and automated coding activity tracking system for online programming classes. Watcher first isolates programming environments for students, which can be immediately utilized for coding activities. Watcher collects and monitors the coding activity of individual students for instructors to provide reports that can detect cheating on programming assignments. A prototype of Watcher served a Unix system programming class in 2020, which included 96 students. Our evaluation results show that Watcher effectively provides various coding activity data to suppress or detect cheating on programming assignments. Furthermore, Watcher offers convenient and fast programming environments by providing the Web-IDE on isolated Linux VMs on the private cloud. We plan to study an automated algorithm that can detect plagiarism by analyzing the data collected from Watcher in various ways in the future. Moreover, we plan to extend Watcher to include more functionalities, such as similarity detection between students’ source code contents in the same class and the provision of meaningful and digested reports from the previously submitted coding activities.

## Figures and Tables

**Table 1:** Comparison of related studies.

	Detect Cheating on Programming Assignments	Independent to Programming Languages	Applicable to the Short Length of Programs	Cloud-Based Environments	Isolated VM per Student	IDE Support
Watcher	✓	✓	✓	✓	✓	Web-based
DevActRec [16]	✗	✓	✗	✗	✗	IntelliJ IDEA, Android Studio
BPlag [12]	✓	✗	✗	✗	✗	None
DSlab [20]	✗	N/A	✓	✓	✗	Web-based
[15]	✗	✓	✓	✗	✗	Eclipse
Hellas et al. [19]	✓	✗	✗	✗	✗	None
MLcite [14]	✓	✓	✗	✗	✗	None
[18]	✓	✗	✗	✗	✗	None
Dolos [13]	✓	✓	✗	✗	✗	None
[17]	✗	✓	✓	✗	✗	Eclipse

**Table 2:** Summarized results of Watcher coding activity. data.

Student	HIGH SCORING STUDENTS				LOW SCORING STUDENTS			
Category	A	B	C	AVG	D	E	F	AVG
# of activities	395	753	972	706.67	9	64	10	27.67
working time (hour)	153.67	40.71	18.57	70.98	16.47	1	2.29	6.46
min file diff. (bytes)	−1314	−5828	−9708	−5616.67	−5535	−3012	−2509	−3685.33
max file diff. (bytes)	1315	5828	9706	5616.33	1300	2868	2516	2228.00
AVG. of file diff (bytes)	0.50	0.00	−1.00	−0.17	−2117.50	−72.00	3.50	−728.67
Final size of source code (bytes)	6094	5444	10,134	7224.00	2210	3116	2516	2614.00

## Data Availability

Not applicable.

## Abbreviations

    The following abbreviations are used in this manuscript:

COVID-19	Coronavirus disease of 2019
CPU	Central processing unit
SW	Software
Student ID	Student identification
IP	Internet protocol
I/O	Input and output
